# Barriers to Cooperation Aid Ideological Rigidity and Threaten Societal Collapse

**DOI:** 10.1371/journal.pcbi.1003618

**Published:** 2014-05-08

**Authors:** Marko Jusup, Tadasu Matsuo, Yoh Iwasa

**Affiliations:** 1 Department of Biology, Kyushu University, Fukuoka, Japan; 2 Faculty of Economics, Ritsumeikan University, Kusatsu, Japan; ETH Zürich, Switzerland

## Abstract

Understanding the factors that promote, disrupt, or shape the nature of cooperation is one of the main tasks of evolutionary biology. Here, we focus on attitudes and beliefs supportive of in-group favoritism and strict adherence to moral consensus, collectively known as ideological rigidity, that have been linked with both ends of the political spectrum. The presence among the political right and the left is likely to make ideological rigidity a major determinant of the political discourse with an important social function. To better understand this function, we equip the indirect reciprocity framework – widely used to explain evaluation-mediated social cooperation – with multiple stylized value systems, each corresponding to the different degree of ideological rigidity. By running game theoretical simulations, we observe the competitive evolution of these systems, map conditions that lead to more ideologically rigid societies, and identify potentially disastrous outcomes. In particular, we uncover that barriers to cooperation aid ideological rigidity. The society may even polarize to the extent where social parasites overrun the population and cause the complete collapse of the social structure. These results have implications for lawmakers globally, warning against restrictive or protectionist policies.

## Author Summary

Attitudes, beliefs, and resulting value systems may represent important motivational and decision-making factors that have strong impact on cooperation in a society. Accordingly, understanding the social function of value systems is a topic of great interest in evolutionary biology, but one where progress is made difficult by the sheer complexity of values-inspired behaviors. Here, we argue that considerable theoretical progress can be made within the indirect reciprocity framework. We show in the context of indirect reciprocity how to construct stylized value systems from a mathematically formalized notion of ideological rigidity. Our simulations indicate that politically imposed restrictions and protectionism favor the evolution of ideologically rigid value systems. The complete collapse of cooperation also arises as a possible evolutionary outcome.

## Introduction

Factors affecting cooperation in a society, such as attitudes, beliefs, and resulting value systems, are a subject of major interest in evolutionary biology. Some examples of considerable importance are in-group favoritism, nationalism, ethnocentrism, intolerance for dissent, submission to strong leadership, and support for tight control all of which were originally linked with the political right [1]. However, evidence was presented to dispel such a link and argue that the same set of attitudes, by serving both the right and the left, is indicative of ideological rigidity rather than a position on the political spectrum [2]. The alleged presence on both ends of the spectrum is likely to make ideological rigidity a potent force in directing the political discourse and ultimately shaping societies. Here, we set to investigate the social function of ideological rigidity, starting from a motivational premise that indirect reciprocity – a cooperation maintaining mechanism based on the evaluation of the reputation [3] – provides a proper framework for our investigation. To establish this premise, we emphasize the dual nature of the aforementioned attitudes and beliefs. On the one hand, ideologically rigid believe in the supremacy of one's group or, at least, distrust anyone who is not a member of this group. Such a belief, broadly termed in-group favoritism, represents an attractive phenomenon for the studies on indirect reciprocity [4]–[6]. On the other hand, ideological rigidity is linked with attitudes that demand strict adherence to moral consensus. Corresponding ideas are again found in the indirect reciprocity framework, where social norms subjected to the evolutionary competition [7], [8] handle dissent from moral consensus in different ways. Our aim is to unify these ideas by incorporating the dual nature of ideological rigidity into stylized value systems and then examine the consequent evolutionary dynamics.

As the first step forward, we formalize the notion of ideological rigidity within the indirect reciprocity framework. In an indirect reciprocity game, members of a society, or players, encounter each other randomly, whereupon one player takes the role of a donor, while the other acts as a recipient. The donor can choose between two actions contingent on the recipient's reputation. By cooperating with the recipient, the donor incurs a cost 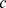, but the recipient benefits from a payoff 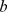 for a net gain of 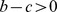 for the society. By defecting, the donor avoids the cost 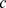, the recipient gains nothing, and the society is exactly where it was before the encounter. Every action is scrutinized by observers who assign the donor an appropriate reputation for the next round of the game. Maintaining a favorable reputation improves the prospects of receiving the payoff 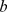 afterwards, thus justifying the willingness to incur the cost 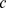 in the first place. The payoff 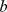 is potentially received in the next round of the game from a third player – hence the name indirect reciprocity – who then serves as a donor, while the current donor takes the role of a recipient. The detailed rules governing which action should be taken and how the reputation should be assessed are called action-assessment strategies and represent a stylized version of the donor's value system. Action-assessment strategies are a part of the central process called the reputation dynamics (see Methods). For now, it is critical that the reputation dynamics can incorporate several action-assessment strategies, allowing us to distinguish between player types and place them appropriately on a scale of ideological rigidity.

The first key aspect of any action-assessment strategy, as the name suggests, is the action rule. We focus on the situation in which all players are discriminators, meaning that donors cooperate only with recipients who have a favorable reputation [9]. Because the action rule is the same for all players, making a distinction between player types requires other key aspects of action-assessment strategies to be more elaborate. One such aspect is the reputation assessment rule. Assessment rules are theoretical representations of social norms that govern the decision-making process of observers while assigning the reputation to donors for the next round of the game. We assume that information spreads from observers to other players rapidly (e.g. through gossip). Two assessment rules are considered. The first of the two rules is called simple-standing or the Sugden rule [10]–[12]. It stipulates that a favorable reputation is assigned to a donor who cooperates with a recipient of favorable reputation or defects from a recipient of unfavorable reputation. An unfavorable reputation is assigned to a donor who defects from a recipient of favorable reputation. Importantly, a favorable reputation is assigned to a donor who cooperates with a recipient of unfavorable reputation, indicating that the Sugden rule liberally follows moral consensus. By contrast, the second of the two rules, called stern-judging or the Kandori rule [12], [13] strictly enforces moral consensus. Cooperation with a recipient of unfavorable reputation leads to an unfavorable reputation assignment for the donor. For an easy comparison, both assessment rules are summarized in Table 1. These concepts are defined in a strict mathematical manner in the section on the reputation dynamics (see Methods).

**Table 1:** The assessment rule.

Observer's type	Sugden		Kandori	
Recipient's reputation	Favorable	Unfavorable	Favorable	Unfavorable
Cooperation	1	1	1	0
Defection	0	1	0	1

Values represent the probabilities that the donor is evaluated favorably conditional on all relevant circumstances (the observer's type, the recipient's reputation, and the donor's action).

Before introducing another key aspect of an action-assessment strategy, we make the assumption that the society consists of two separate parts. Namely, an inner circle (e.g. a nation state) is embedded into a much larger outer world (e.g. the international community), where the cooperation between the two parts of the society, though allowed, is made difficult (e.g. by the national border control). Members of the inner circle (i.e. insiders) thus have a high probability, denoted 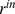, of encountering other insiders, but only a small probability, 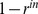, of meeting a player from the outside world (i.e. an outsider). For an insider, cooperation with the outside world also carries an additional cost, 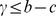 (e.g. a tariff). Herein, we are primarily interested in the evolutionary dynamics (see Methods) of value systems inside the inner circle and the subsequent implications for ideological rigidity of insiders.

The division of the society into two separate parts leads us naturally to another key aspect of an action-assessment strategy. Within the inner circle, because encounters with the outside world are rare, it is fairly reasonable to presume that distrust towards outsiders can take root among a fraction of the insiders. These insiders exhibit strong in-group favoritism in the sense that all cooperation with outsiders is suspended and no benefits from the outside world are accepted. The remaining insiders, by contrast, reject in-group favoritism, maintain cooperation with the outside world, and receive the accompanying benefits. The setting we describe here is not without a historical precedent. A resemblance can be found in pre-modern Japan [6], where the two dominant value systems, one called bushido (the way of warriors) and the other called shonindo (the way of merchants), held opposing positions on in-group favoritism. In Western culture, many parallels can be drawn by examining the differences between the Maghribi and the Genoese [14]. However, being primarily motivated by the bushido-shonindo dichotomy, we name the fraction of the insiders that embrace in-group favoritism “bushi”. The remaining insiders that reject in-group favoritism are named “shonin”.

The two introduced aspects of action-assessment strategies (Sugden vs. Kandori and shonin vs. bushi) allow us to distinguish four types of insiders. Sugden-shonin (hereafter Ss) liberally follow moral consensus, reject in-group favoritism, and hence are considered ideologically non-rigid. A step up on the scale of ideological rigidity are Kandori-shonin (Ks), who strictly enforce moral consensus, yet reject in-group favoritism. Sugden-bushi (Sb), while liberal towards moral consensus, endorse in-group favoritism. We consider the stance of Sb players to be more ideologically rigid than the stance of Ks players because in-group favoritism as defined herein limits the scope of cooperation far more strongly than the strict enforcement of moral consensus. The most ideologically rigid are Kandori-bushi (Kb), who enforce moral consensus and embrace in-group favoritism. Besides these four types of players we entertain the notion of social parasites in the form of unconditional defectors (often denoted AllD in the literature, hereafter simply Ad). For the outside world, which is much bigger than the inner circle, interactions with insiders are inconsequential. Modeling the evolutionary dynamics (see Methods) of value systems in the outside world is possible using the same mathematical framework as for the inner circle, but with the probability of an outsider meeting another outsider set to unity. The inner circle is, therefore, a set of measure zero. Because we are interested in the evolutionary dynamics of value systems in the inner circle, the outside world is assumed to be in a stable equilibrium populated only by Ss or Ks players. Such a simple structure of the outside world can be justified by the fact that any other more complex structure would only diminish the benefits from cross-border encounters which is qualitatively captured by increasing the value of the parameter 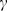.

## Results and Discussion

**Figure 1:**
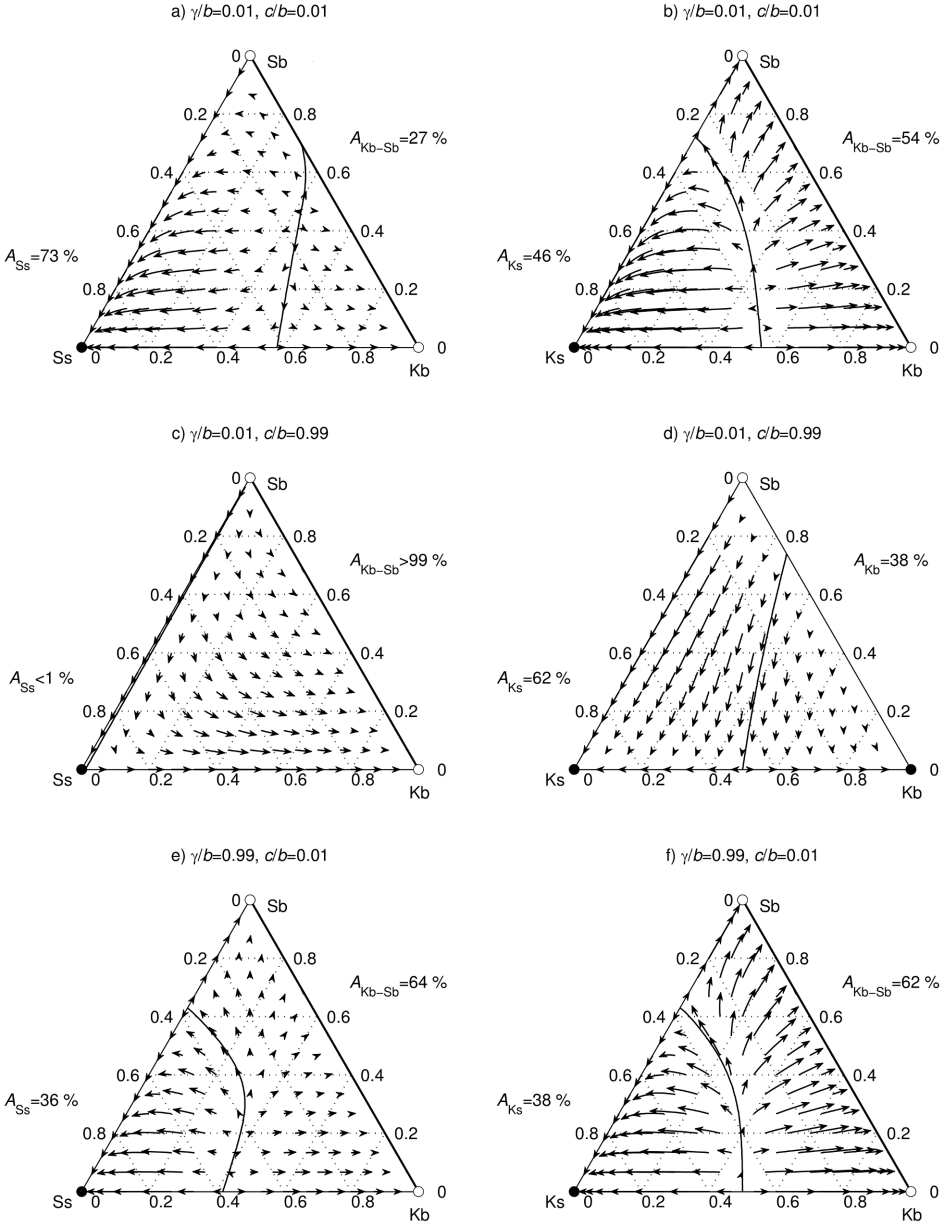
Performance of action-assessment strategies. (a) Low barriers to cooperation make the Ss strategy successful against Kb and Sb strategies. (b) Under the same conditions as in (a), the Ks strategy performs worse than the Ss strategy. (c) High barriers to cooperation are detrimental for the Ss strategy. (d) Under the same conditions as in (c) the Ks strategy is successful against Kb and Sb strategies. (e) The success of the Ss strategy in (a) diminishes when the cost of cross-border cooperation is high. (f) Under the same conditions as in (e) the Ks strategy is affected less than the Ss strategy.

**Figure 2:**
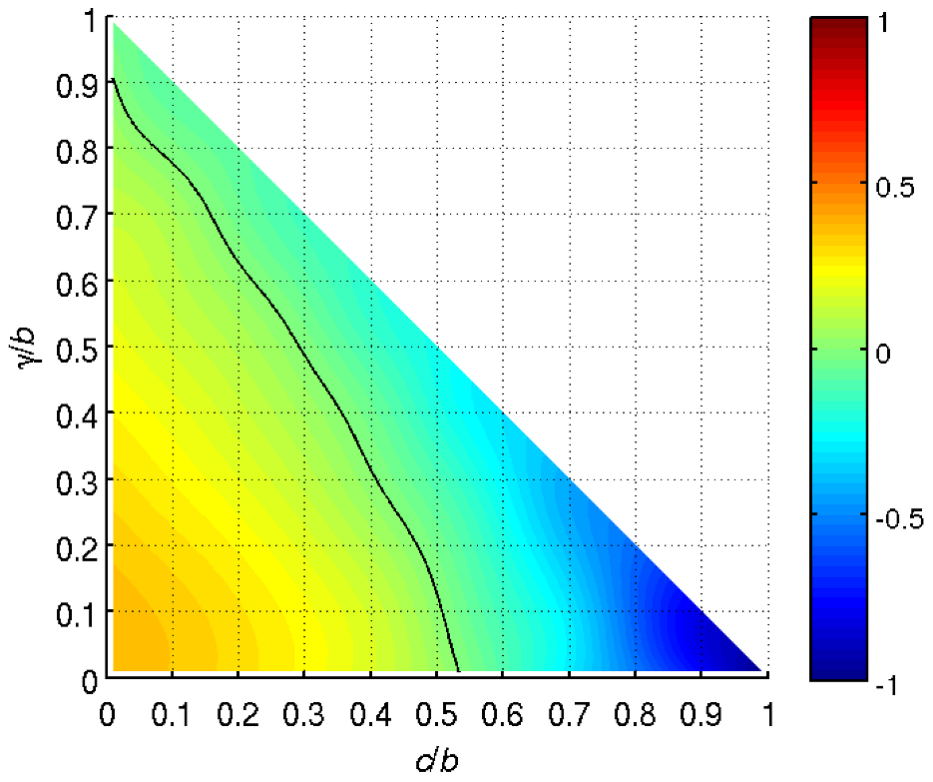
A continuous map of the parameter space. Relative performance of Ss and Ks strategies, 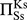, is shown for all reasonable cost-benefit ratios. The black curve approximates the border at which both strategies are equally effective, i.e. 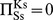.

**Figure 3:**
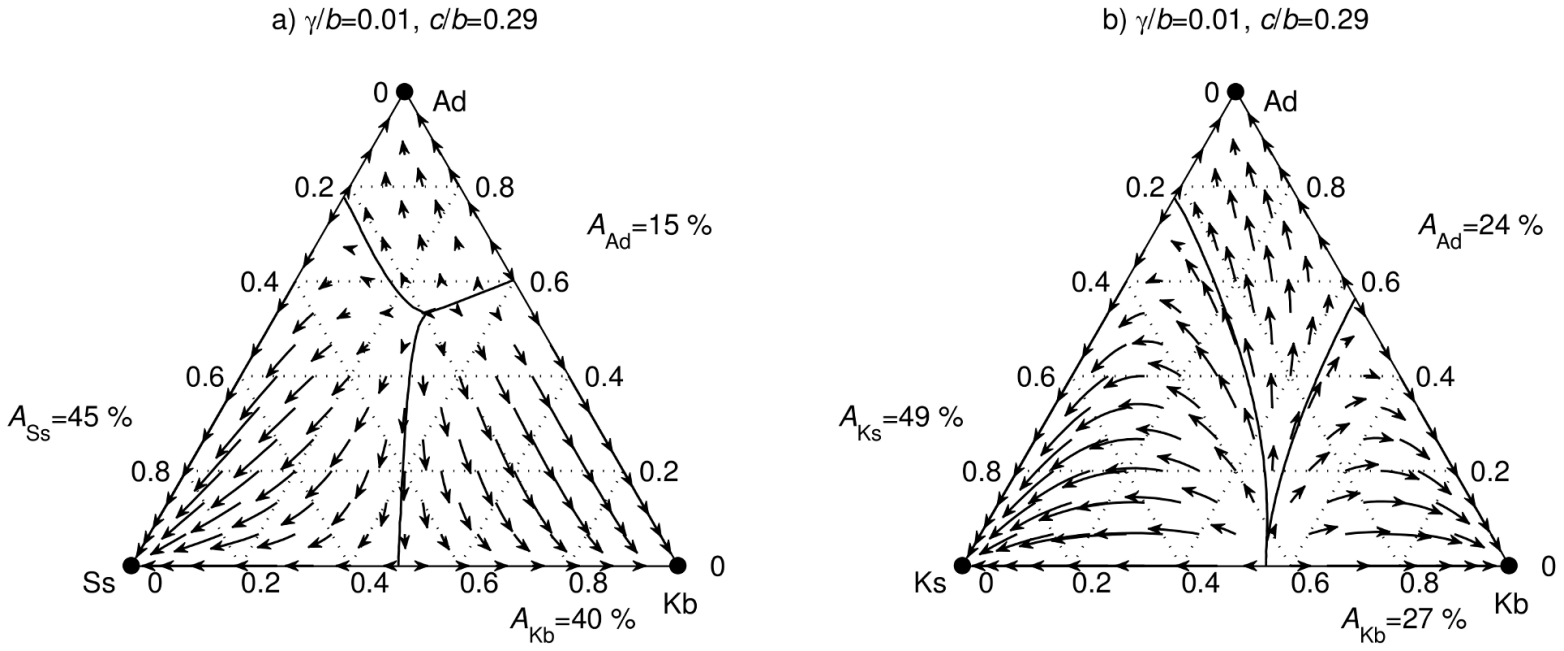
Ideological rigidity and the collapse of social structure. (a) Social parasites cannot invade the inner circle dominated by any combination of ideologically non-rigid Ss and rigid Kb players. (b) Replacing ideologically non-rigid Ss with more rigid Ks players may polarize the inner circle to the extent where social parasites uncontrollably spread out and eventually suppress cooperation.

We explore the dual nature of ideological rigidity and its social function by means of indirect reciprocity games. Differences in adherence to moral consensus are reflected in the performance of the more liberal Sugden against the stricter Kandori rule (S and K in shorthand notation, respectively). Similarly, opposite attitudes towards in-group favoritism are reflected in the performance of open-minded shonin against distrustful bushi players (s and b, respectively). The focus is placed on the most illustrative cases, meaning a relatively closed inner circle 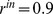 in which the set of possible player types is either 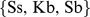 or 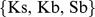. Though the model can handle any number of player types, having three types per simulation permits effective visualization and comparison of the results. We start with a technical description of two opposing situations, one where barriers to cooperation are low and the other where barriers are high (Fig. 1). To achieve this, we set both cost-benefit ratios, 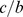 and 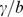, close to zero and subsequently increase either one towards unity. The results are then generalized by continuous mapping of the parameter space (Fig. 2) and finally by inclusion of social parasites (Ad) into simulations (Fig. 3).

Low barriers to cooperation favor ideologically non-rigid Ss strategy (Figs. 1a, b). From ternary plots it is apparent that the vertices Ss and Ks share the property of being locally stable monomorphic attractors. By contrast, neither Kb nor Sb vertices have this property, but rather the whole segment connecting them is a locally stable dimorphic attractor (the Kb-Sb attractor). Comparing the sizes of the corresponding domains of attraction reveals the evolutionarily advantageous action-assessment strategy. When cost-benefit ratios are close to zero (Fig. 1a), the Ss attractor not only overshadows the Kb-Sb attractor in terms of the size of the domain of attraction (73% vs. 27%), but a rare occurrence of Ss players in an inner circle dominated by Sb players leads to a successful invasion. Under the same conditions (Fig. 1b), the Ks attractor fares less well, commanding a smaller domain of attraction than the Kb-Sb attractor (46% vs. 54%) and failing to successfully invade the inner circle dominated by any combination of Kb and Sb players.

Increasing barriers to cooperation make the ideologically non-rigid Ss strategy evolutionarily disadvantageous (Figs. 1c, d). For a society to maintain feasible cross-border interactions, ideological non-rigidity needs to be abandoned in favor of a more ideologically rigid Ks strategy. Namely, when the cost-benefit ratios 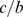 is set close to unity, the domain of attraction of the Ss attractor (Fig. 1c) is greatly reduced in favor of the Kb-Sb attractor (<1% vs. >99%) despite Ss players still being able to invade an Sb-dominated inner circle. By contrast, the Ks attractor fares much better than originally (Fig. 1d). Its domain of attraction is now larger than that of the alternative (locally stable monomorphic) Kb attractor (62% vs. 38%) and a rare occurrence of Ks players in an Sb-dominated inner circle leads to a successful invasion. It is worth emphasizing that vulnerability to invasion by both Ss and Ks strategies makes the Sb strategy a weak candidate for the ideologically rigid.

The increasing cost of cross-border interactions aids ideological rigidity (Figs. 1e, f). The effect is twofold because the Sb strategy turns evolutionarily viable and the Ks strategy gains an (albeit marginal) evolutionary advantage over the Ss strategy. When the ratio 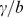 is set close to unity and 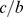 is kept near zero, the benefit of encountering outsiders is reduced and, therefore, Ss and Ks strategies are negatively impacted. Accordingly, the inner circle dominated by Sb players can no longer be invaded by either Ss or Ks players. The domains of attraction of Ss and Ks attractors become smaller than that of the Kb-Sb attractor (36% vs. 64% and 38% vs. 62%, respectively). A notable difference between Ss and Ks strategies, however, is that the negative impact of high 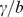 is worse for the former (Figs. 1a, e) than the latter (Figs. 1b, f) strategy. When 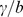 is close to unity the domain of attraction of the Ss attractor is reduced to half its original size (from 73% to 36%), whereas the reduction for the Ks attractor is minimal (from 46% to 38%).

The three extreme cases examined heretofore are illustrative, but not exhaustive because any combination of cost-benefit ratios that satisfies 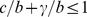 is reasonable. Consequently, we map the parameter space in a continuous manner, focusing in particular on the relative performance of Ss and Ks strategies. We emphasize the relative performance because the evolutionary advantage of one strategy over the other changes with the location in the parameter space. By comparison, vulnerability to invasion suggests that for the ideologically more rigid the Sb strategy is a weak alternative. To summarize the relative performance of the two strategies over a wide range of cost-benefit ratios, we denote respectively by 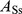 and 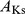 areas of the domains of attraction corresponding to Ss and Ks attractors and introduce a performance indicator 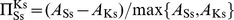. Note that 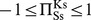, where positive (negative) values indicate the evolutionary advantage of Ss (Ks) players. The simulation results (Fig. 2) confirm the notion that Ss players have an evolutionary advantage when barriers to cooperation are low; that is, when the sum of the two cost-benefit ratios is close to zero. As barriers become higher, the Ks strategy turns out to be advantageous. Particularly detrimental for Ss players is the increase in the cost-benefit ratio 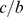 because as it approaches unity the area 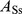 tends to zero. Increasing 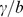 affects both Ss and Ks players negatively, but the area 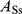 is much more sensitive to the change in 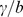 than 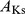.

Pursuing a technical description of the simulation results so far sheds new light on the evolution of value systems as implied by the indirect reciprocity framework, but remains silent on the underlying mechanisms. We are thus required to make an extra effort to access these mechanisms and in return gain an intuitive grasp of the mathematical formalism being applied. Starting from a comparison of bushi and shonin players, the former are at a fundamental disadvantage because of refusing to interact with the outsiders. Such a situation is exemplified by the success of Ss over Sb players in Fig. 1a. Bushi, in fact, may not represent an evolutionarily viable alternative at all without sufficiently closed borders (i.e. 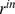 slightly below unity). If this criterion is met, the difference between Sugden and Kandori rules plays a major role. Players adopting the Sugden rule, due to their liberal stance, receive benefits and incur costs of cooperation more often than players adopting the Kandori rule – a clear advantage when 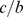 is low. As 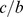 approaches unity, the fortunes reverse. We can now understand why Sb players can resist Ks players in Fig. 1b, as well as the success of Kandori players in Figs. 1c and d. Protectionism (i.e. 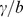 slightly below unity) naturally helps bushi agenda, which is best illustrated by how Ss players lose their advantage over Sb players as the cost of cross-border interactions increases (Figs. 1a, e). The same effect is visible by comparing Figs. 1b and f, although it is much weaker because the Sugden rule to a certain extent shields Sb against Kb players even when the cost of cross-border cooperation is low. The described mechanisms can be used to explain all intermediate outcomes in Fig. 2.

Having determined that the Kb strategy shields the ideologically rigid from invasion, while the ideologically less rigid should choose between Ss and Ks strategies contingent on how high barriers to cooperation are set, we consider the effect of social parasites on the society. Indirect reciprocity games with two sets of possible player types, 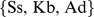 and 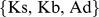, reveal important qualitative differences (Fig. 3). With moderately low barriers to cooperation, the conflict between Ss and Kb strategies in the presence of social parasites results in three distinct domains of attraction accompanied with three locally stable monomorphic attractors, as well as three dimorphic and one trimorphic equilibria (Fig. 3a). Though the size of each domain of attraction is parameter-dependent, the remarkable outcome is that there are no openings for an invasion. By contrast, the conflict between Ks and Kb strategies in the presence of social parasites lacks a trimorphic equilibrium and leaves the dimorphic equilibrium of Ks and Kb players vulnerable to invasion in the case of a rare occurrence of Ad players (Fig. 3b). Therefore, by making the ideologically more rigid Ks strategy evolutionarily advantageous over the Ss strategy, rising barriers to cooperation not only aid ideological rigidity, they even threaten the collapse of the social structure.

Looking at the results in Fig. 3, what we truly observe are the negative consequences of the maxim “the enemy of my enemy is my friend” as well as the way to avoid these consequences. When the inner circle is populated with an ideologically more rigid combination of Ks and Kb (along with Ad) players, even if they initially treat each other favorably, after a while a Ks player will cooperate with an outsider and be assigned an unfavorable reputation by the Kb observer. Such a player is bound to be denied cooperation from a Kb donor, resulting in an unfavorable reputation assignment for this donor from the Ks observer. A rift between Ks and Kb players forms. Ad players may eventually take the advantage of such a rift (Fig. 3b) because when they defect from a Ks recipient they receive benefits from a Kb donor and vice versa. Note that with stern-judging the rift only widens after every interaction of either a Ks or a Kb donor with an Ad recipient. The reason is that the Ad recipient is treated favorably by one side and unfavorably by the other, resulting with certainty in an unfavorable reputation assignment for the donor. Replacing Ks players with ideologically non-rigid Ss players does not prevent the rift from opening. Ss players, however, mend the rift after a Kb donor cooperates with an Ad recipient by assigning a favorable reputation to this donor. Liberal attitude towards moral consensus thus makes it impossible for social parasites to invade the inner circle (Fig. 3a) and can be seen as a layer of stability for the social structure. To confirm the robustness of the described mechanisms, we performed simulations (results not shown) with two additional sets of possible player types: 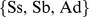 and 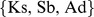. It turned out that only the ideologically rigid combination of Ks and Kb players was vulnerable to an invasion by Ad, agreeing with the notion that liberal attitude towards moral consensus had a stabilizing effect on the society. In the context of the model robustness, we did not simulate unconditional cooperators nor the first-order scoring rule because it was shown that neither could maintain stable cooperation [8]; all else being equal, the former got eliminated in the presence of Sugden, Kandori, and Ad, whereas the latter, if not eliminated, became indistinguishable from Ad. The presence of unconditional cooperators, nonetheless, might have favored Kandori over Sugden to a certain extent because the Sugden rule would have encouraged more cooperativeness and hence higher costs in comparison with the Kandori rule [8].

In the modern world, two omnipresent processes affecting barriers to cooperation are the technological development (lowering 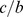) and globalization (lowering 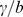). Because both of these processes make cooperation easier, our results imply (to the extent game theoretical representations are valid in a complex reality) that the modern world is conducive of ideologically non-rigid societies with presumably an increasing number of functioning democracies and more economic liberties [15]. Support can be found in indisputable growth of electoral democracy among the world's nations, especially over the past three decades, although the Democracy Index compiled by the Economist Intelligence Unit suggests that the overall quality of democracy is stagnating since the financial crisis of 2007–2008. As for economic liberties, the average Economic Freedom of the World Index reported by the Fraser Institute indicates steady increase from 1980 until 2006, but again a period of stagnation during the 2008–2012 global recession. Looking at our results from a different angle, a remarkable implication is that restrictive or protectionist policies aid the creation of ideologically rigid societies. Perhaps then it is not surprising that the above indices are stagnating in the midst of a five-years long recession. This is, after all, the second worst economic contraction since the Great Depression of the 1930s, which itself brought on a number of restrictive or protectionist policies, coinciding with the rise of multiple totalitarian regimes and ending only after the deadliest conflict in the human history.

Analyzing the social role of ideological rigidity within the indirect reciprocity framework, we uncovered evolutionary outcomes that warn against restrictive or protectionist government policies. Yet to prevent from falling into the trap of naive rationalism or worse interventionism, a constant remainder is needed that these outcomes follow from a mathematically tractable representation of immensely complex human concepts such as value systems. We, therefore, perceive the present and similar studies as theoretical constructs that identify the potential, rather than the actual, drivers of social phenomena. Keeping this important distinction in mind does not diminish the multitude of opportunities for the field. Our understanding of the factors that promote (e.g. punishment), disrupt (e.g. corruption) or shape the nature (e.g. spontaneous in-group favoritism) of cooperation is still quite limited.

## Methods

### Reputation dynamics

The reputation dynamics control intra-generational partitioning of players according to their reputation. Intuitively, the outcomes of the reputation dynamics specify probabilities that the generation of players of a particular type will be assigned a particular reputation. More formally, we are concerned with a discrete probability measure defined on a sample set, 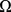, where the sample set is built from two basic constituents, the set of player types, 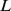, and the set of all possible reputations, 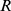. Because five distinct player types are considered, the set 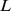 could generally be any combination of 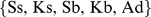. It is beneficial, however, to display the results of extensive numerical simulations on ternary plots by referring only to the most illustrative outcomes. The main reason is that handling three player types at once permits us to effectively visualize and compare the results. The set 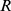 is accordingly limited to 3-combinations with repetitions (or 3-multisets) of the set 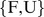, where F and U denote a favorable and an unfavorable reputation, respectively. Hence, 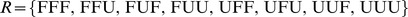. With these basic constituents, the sample set is given by the Cartesian product of the form 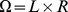, so that 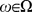 implies 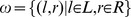. The discrete probability measure of concern, denoted by 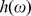 because it is closely related to the so-called honor score [16], is fully defined by specifying how it operates on the elements of 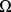. To emphasize the dependence on the type and reputation of players, we introduce a short-hand notation 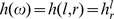. The letters 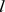 and 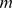 are used throughout the text to denote player types, primarily that of a donor and a recipient, respectively. The letters 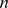 and 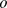 are reserved for the type of observers and outsiders, respectively. It is also useful to reserve the letters 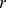 and 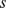 for the reputation of donors and recipients, respectively. Note that 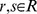 and, therefore, stand for three different reputations. When interested only in the reputation from the viewpoint of type 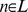 observers, we can write 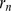 and 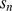. In addition, to keep formulas for the probabilities 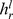 more tractable, it is helpful to introduce two auxiliary functions as shown henceforth.

The first of the two auxiliary functions, denoted 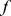, is called the action rule. Because each interaction in the game involves two players, a donor and a recipient, the action rule specifies the probability of an action 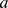 being undertaken by the type 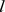 donor towards the recipient with the reputation 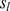 from the donor's viewpoint. Only two actions are possible, so that 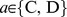, where C and D stand for cooperation and defection, respectively. It follows, 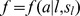. For 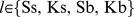 the action rule is
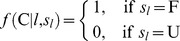
and 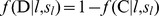. Ad players never cooperate, thus 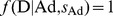.

The second auxiliary function, denoted 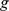, is called the assessment rule. Because observers assign new reputations to donors after every interaction, the assessment rule represents the probability that the donor will be assigned the reputation 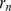 by the type 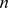 observer if the recipient's reputation from the observer's viewpoint is 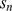 and an action 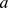 is taken. Consequently, 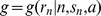. The case 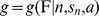 is presented in Table 1, where the distinction between 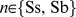 and 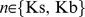 formally defines Sugden and Kandori observers. Similarly with the action rule, 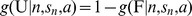. Ad players treat every donor unfavorably, thus 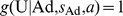.

By combining action and assessment rules, we can formally express how action-assessment strategies determine the probabilities that the donor will be assigned the reputation 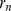 conditional on all relevant circumstances. Such conditional probabilities are crucial for calculating the probabilities 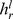. If the recipient is an insider, the relevant circumstances are specified by the type of observer (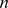) and donor (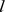), as well as the recipient's reputation from observer's (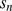) and donor's (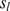) viewpoints. Accordingly, we introduce
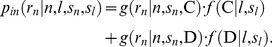
(1)


If the recipient is an outsider, both action and assessment differ between shonin and bushi players, thus providing a way to formalize the distinction between the two. The former players make an effort to learn the outsider's reputation, whereas the latter simply dismiss the outsider as a player with an unfavorable reputation. Such a situation implies that outside recipients, who are by assumption of type 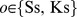, are perceived favorably by the observer and the donor, i.e. 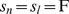, if and only if 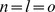. Consequently, we can introduce the probability 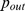 as an analogue to 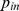 conditional only on the type of observer (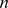), donor (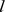), and outsider (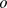) by
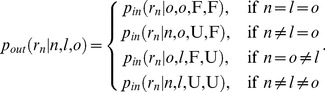
(2)


We mentioned that the reputation dynamics controlled partitioning of players according to their reputation within a generation. Therefore, each generation plays many rounds of the game, whereby every player serves both as a donor and as a recipient once per round. When serving as a donor, the player encounters either an insider recipient with probability 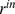 or an outsider recipient with probability 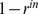. If the recipient is an insider, the probability of it being a player of type 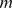 is 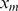 and the probability of its reputation being 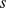 is 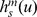, where 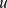 denotes the current round of the game. Using Eqs. (1) and (2), the probability that the type 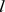 donor is assigned the reputation 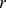 for the next round, 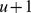, becomes
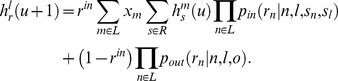
(3)After (infinitely) many rounds of the game, the probabilities 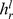 converge to the equilibrium values defined by 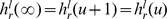. These equilibrium values are then used to simulate the evolutionary dynamics of value systems inside the inner circle.

### Evolutionary dynamics

The evolutionary dynamics of value systems inside the inner circle is modeled using the replicator equations. If we denote the fraction of the type 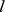 players at the generational time 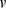 with 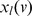, so that 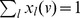, then the same fraction in the next generation, 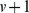, is given by
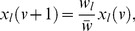
(4)where 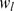 is the fitness of the type 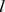 players and 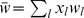 is the average fitness (both at time 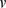). Fitness is a function of the equilibrium probabilities 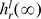 (and the parameters 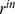, 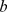, 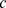, and 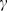) because players of a given type receive the payoff 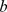 to the extent they are perceived favorably by their respective donors and incur the cost 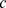 to the extent they perceive their recipients favorably. Assuming that outsiders are of type 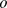, i.e. 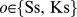, the above considerations can be written in general mathematical terms as
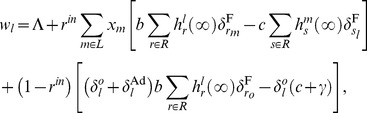
where 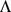 is an arbitrary basic level of fitness and 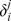 is the usual Kronecker delta symbol. Besides 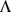, two additional summands appear in the last equation. The first of the two summands represents the difference between the benefits received and the costs incurred from within-group encounters. Note that the benefits from type 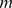 players are received only if the reputation 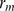 is favorable, i.e. 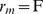. Similarly, when encountering type 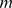 players the costs are incurred only if the reputation 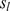 is favorable, i.e. 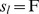. The second of the two summands also represents the difference between benefits and costs, but now as a result of cross-border encounters. Here, insiders who are of the same type as outsiders, i.e. when 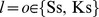, receive the benefits if their reputation is favorable (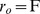) and incur the cost 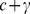 with certainty because the outside world is assumed to be in a stable equilibrium populated by the type 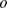 players. Social parasites behave opportunistically in the sense that they receive the benefits whenever their reputation is favorable from the viewpoint of the type 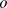 observer.

### Implementation

We performed numerical simulations based on the described methodology to (i) visualize convergence of the model over the generational time scale, (ii) delineate the domains of attraction, and (iii) estimate their sizes. We achieved these goals in several steps. First, we defined a grid with 5050 points distributed uniformly over the ternary domain 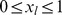, 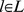, and 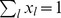. Coordinates of each grid point served as the initial conditions for one model run. Every run consisted of many generational time steps, where in a single step multiple rounds of the game played out according to Eq. (3). Instead of presetting the number of rounds, we waited until the difference 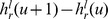 reached the desired accuracy. The resulting approximation of the equilibrium probabilities 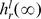 allowed us to calculate the fitness of all player types and advance their respective fractions, 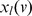, into the next generation, 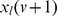, using Eq. (4). Eventually, the model converged to one of the attractors, forming a link between the starting point and the attractor. In the second step, we chose a uniform subset (134 points) of the initial grid for which sample paths over the generational time 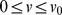 were stored and subsequently visualized in the ternary plots (Figs. 1 and 3). For the visualization, we used curved arrows to characterize the direction and the rate of convergence along sample paths; the longer an arrow, the faster the convergence rate along that particular path. The third step began after completing all 5050 runs for a fixed parameter set. Because every grid point had been linked with an attractor, we could isolate the neighboring points that belonged to two different domains of attraction. Segments connecting such neighboring points were further subdivided with five equidistant points to provide the initial conditions for extra runs in which an even closer pair belonging to two different domains of attraction could be determined. The process continued until the distance between the neighboring points reached the desired accuracy and thus revealed the location of the border between the adjacent domains of attraction. In the final step, we calculated the fraction of grid points linked to each attractor as an estimate of the size of the corresponding domain of attraction.
